# The past, present, and future of immunization in the Americas

**DOI:** 10.26633/RPSP.2017.121

**Published:** 2017-12-20

**Authors:** Jon Kim Andrus, Ananda Sankar Bandyopadhyay, M. Carolina Danovaro-Holliday, Vance Dietz, Carla Domingues, Figueroa J. Peter, Posenato Garcia Leila, Alan Hinman, Mirta Roses, Cuauhtémoc Ruiz Matus, Jose Ignácio Santos, Fred Were

**Affiliations:** 1 Adjoint Professor and Director of the Division of Vaccines and Immunization Center for Global Health, University of Colorado Colorado United States Adjoint Professor and Director of the Division of Vaccines and Immunization, Center for Global Health, University of Colorado, Colorado, United States.; 2 Senior Program Officer Polio, Global Development, The Bill & Melinda Gates Foundation Seattle United States Senior Program Officer, Polio, Global Development, The Bill & Melinda Gates Foundation, Seattle, United States.; 3 Scientist, Global Immunization Monitoring and Surveillance Group World Health Organization Geneva Switzerland Scientist, Global Immunization Monitoring and Surveillance Group, World Health Organization, Geneva, Switzerland.; 4 Immunization Services Division Centers for Disease Control and Prevention Atlanta United States Immunization Services Division, Centers for Disease Control and Prevention, Atlanta, United States.; 5 Coordenadora Geral do Programa Nacional de Imunizações Coordenadora Geral do Programa Nacional de Imunizações Brasilia Brazil Coordenadora Geral do Programa Nacional de Imunizações, Brasilia, Brazil.; 6 TAG Chair, Professor of Public Health, Epidemiology & HIV/AIDS University of the West Indies Kingston Jamaica TAG Chair, Professor of Public Health, Epidemiology & HIV/AIDS, University of the West Indies, Kingston, Jamaica.; 7 Secretariat of Health Surveillance Brazilian Ministry of Health Brasília Brazil Secretariat of Health Surveillance, Brazilian Ministry of Health, Brasília, Brazil; 8 Consulting Senior Advisor, Center for Vaccines Equity Task Force for Global Health Decatur United States Consulting Senior Advisor, Center for Vaccines Equity, Task Force for Global Health, Decatur, United States.; 9 Directora Emérita, Organización Panamericana de la Salud Directora Emérita, Organización Panamericana de la Salud, Buenos Aires Argentina Directora Emérita, Organización Panamericana de la Salud, Buenos Aires, Argentina.; 10 Unit chief, Family Immunization Unit,, Pan American Health Organization Washington, D.C. United States Unit chief, Family Immunization Unit,, Pan American Health Organization, Washington, D.C., United States.; 11 Profesor, Facultad de Medicina de la Universidad Autónoma de México Profesor, Facultad de Medicina de la Universidad Autónoma de México Mexico City Mexico Profesor, Facultad de Medicina de la Universidad Autónoma de México, Mexico City, Mexico; 12 Dean, School of Medicine University of Nairobi Kenyatta National Hospital Campus, Nairobi Nairobi Kenya Dean, School of Medicine, University of Nairobi Kenyatta National Hospital Campus, Nairobi, Kenya

Immunization has come a long way since Englishman Edward Jenner’s discovery in 1796 that cowpox not only protected against smallpox but could also be deliberately transmitted from one person to another as a mechanism of protection. His scientific discovery became a new procedure: “vaccination.” (1) Published in 1798, vaccination was implemented in Spain in 1800 after Emperor Carlos IV’s daughter, Infanta María Luisa, suffered from smallpox. Royal Surgeon Francisco Javier de Balmis y Berenguer (1743–1819), originally from Valencia, Spain, recommended the procedure, having become a staunch supporter of the new vaccine after witnessing the ravages of the smallpox epidemic of 1779 in Mexico, primarily among the indigenous population. His recommendation led to the decision to administer the vaccine to the entire Spanish empire, and set into motion the Royal Philanthropic Vaccine Expedition to take the smallpox vaccine to Spanish colonies overseas (America and the Philippines). (2)

But there was a problem. In vitro, the vaccine lasted only 12 days, but had to be adequately conserved for the months-long journey to the vaccination site. Thus “arm-to-arm” transfer of the vaccinia virus among children aged 4 to 14 years old was developed to address this, and vaccination strategies began to evolve. Children involved in arm-to-arm transfer of vaccinia virus would present only mild symptoms, but were left immune to the more aggressive smallpox virus. Armed with this approach, twenty-two children were selected from the Santiago de Compostela Orphanage, and the expedition sailed out of the port of La Coruña on the María Pinta on 30 November 1803, headed first to the Canary Islands, to carry out the vaccination expedition. On board were the vaccine, surgical and scientific instruments, and a translation of the *Historical and Practical Treatise on Vaccines* by Moreau de Sarthe, which were to be distributed to the vaccination commissions that would be formed at sites where the expedition landed. After landing at the Port of La Guaira, in what it is now Venezuela, Balmis decided to divide the expedition in half: one half, led by José Salvany, administered the vaccine for seven years in Venezuela and the Viceroyalty of Peru (now Panama, Colombia, Ecuador, Peru, Chile, and Bolivia). The second half, led by Balmis himself, administered the vaccine in New Spain (now Mexico). There, he selected an additional 25 orphans to carry the vaccine across the Pacific Ocean aboard the Magallanes. Balmis left the port of Acapulco bound for Manila, in the Philippines, on 8 February 1805, and from there continued on toward Macao and administered the vaccine to people in several cities in Chinese territory, all the way to Canton province. Balmis and Salvany’s expedition (1803–1814) was the first worldwide philanthropic vaccination campaign. Its legacy was the creation of vaccine boards and the recognition of the importance of concerted public health campaigns in preventing and fighting epidemics. (2)

In 1959, the World Health Organization (WHO) adopted a resolution calling for the global eradication of smallpox, and allocated funds specifically for this effort. With smallpox still circulating in some areas of the Americas, Africa, and Asia in the 1960s, WHO launched the Intensified Smallpox Eradication Program in 1967. The last case of smallpox in the Americas was recorded on 19 April 1971 in Brazil, leading to Pan American Health Organization (PAHO) Resolution CD20.23/1971, which announced the United States government’s decision to suspend mandatory smallpox vaccinations and subsequently to Resolution CD22.R17/1973, the historic declaration that the disease had been eliminated in the Americas. The last case in the world was identified in 1977, and by then the idea that a similar approach could be used to increase the administration of other available vaccines had already taken hold. D.A. Henderson, who joined WHO in 1967 during the final battle against smallpox, said: “If a vaccinator in Africa can reach 500 children a day against smallpox, why don’t we do the same with other vaccines?” (3)

WHO Resolution AMS27.57/1974, which established the Expanded Program on Immunization (EPI), did not allocate sufficient resources, but the enthusiasm leading up to the Alma-Ata Conference inspired WHO Director Dr. Halfdan Mahler, in 1977, to allocate $1 million of the regular budget to strengthening the EPI. Later, UNICEF Director James Grant offered his organization’s full support to this endeavor. In 1976, Dr. Ciro de Quadros, a brilliant Brazilian epidemiologist known for helping to eliminate smallpox in his country, and later for being part of the final assault against the virus in Africa, was named PAHO’s regional advisor on immunization. Dr. de Quadros urged every country to establish a national-level immunization program led by a dedicated director. In September 1977, the XXV Session of PAHO’s Directing Council adopted Resolution CD25.27, expanding immunization in the Americas, following recommendations 2, 3, and 5 of the Final Report of the III Special Meeting of Ministers of Health of the Americas, held in Santiago, Chile, from 2 to 9 October 1972, to evaluate the 10-Year Health Plan for the Americas. (4) Recommendation 2 aimed to reduce vaccine-preventable morbidity and mortality from measles, whooping cough, tetanus, diphtheria, and polio through systematic, comprehensive vaccination programs. Recommendation 3 aimed to reduce mortality rates from measles, whooping cough, and tetanus. And recommendation 5 aimed to reduce tuberculosis mortality by combining the BCG vaccine in children under 15 with detection and specialized treatment of patient, using general health services. Resolution CD25.37 included strengthening the cold chain, logistics, and vaccine storage and distribution, and evaluated a proposal for a Revolving Fund to facilitate the procurement of vaccines and related supplies, which was created in 1979. At the Pan American Health Conference of 1982, Resolution CSP21.R7 approved a call for bids to finance the Fund and the creation of the EPI Regional Advisory Committee (now known as the PAHO Technical Advisory Group of Experts (TAG) on Immunization) (5,6)

The implementation of the EPI has helped to catalyze progress in different areas, including:

1.**Vaccination laws.** Since the first Law on Vaccination was passed in Belize in 1963 (7), lawmakers in every country of the Region have made efforts to ensure that their national budgets include free universal vaccine coverage in national programs, following guidelines of the WHO Strategic Advisory Group of Experts (SAGE) on Immunization Global Advisory Committee, the PAHO TAG regional committee, and national immunization committees made up of independent experts known for their integrity, leadership and achievements. Member States also underscore their commitment to vaccination by continuing to declare it as a public good.(8)

2.**Management.**Since the creation of the EPI in the Americas, country ownership has been the guiding principle: periodic meetings of national program directors have been held, and countries have boosted innovation and collective creativity by sharing immunization and vaccine-preventable disease surveillance data, along with their immunization experiences including improvement of the cold chain, information systems, supplies, and program financing.

3.**Revolving Fund.**Demonstrating how countries prioritize immunizations, national immunization programs are in large part, and in the majority of countries, funded by a country’s own resources. The Revolving Fund, an example of comprehensive PAHO technical cooperation for Member States, was created in 1979 and has been one of the pillars for success of self-sustained national immunization programs throughout the Region. The Fund ensures that all vaccine requirements are consolidated (economy of scale) to cover up to 8 million births per year (80% of Latin America and the Caribbean). (9)

4. **New vaccines introduction.**All of this has helped ensure the sustainability of EPI in the Region of the Americas, and its expansion outward from a child-only vaccination focus, with vaccines that only target 6 diseases to immunization for entire families with vaccines that target more than 15 diseases including *Haemophilus influenzae* type b, hepatitis B, mumps, rubella, influenza, yellow fever, pneumococcus, rotavirus and human papilloma virus.

5. **Immunization policies.** Formulation of immunization policy on a global level can be noted in the implementation of a series of large and complex polio vaccine studies. The clinical evidence-base generated from these studies has contributed to the global policy formulation for the polio endgame. (10–15)

To commemorate the 40th anniversary of EPI in the Americas, this supplement of the *Pan American Journal of Public Health* summarizes the past, provides a critical analysis of the present, and offers new perspectives on future challenges and opportunities. The call for specific articles was well received: original articles, reviews, opinion pieces, and special reports were submitted reflecting regional, subregional, and country-specific perspectives. This edition opens with a remembrance and recognition of the legacy of Dr. de Quadros, who was an inspiring leader of immunization in the Americas and around the world. Much of the success of the EPI in the Americas must be attributed to his creative and dynamic leadership over three decades. Several articles emphasize countries’ ownership of their immunization programs and the Region’s pioneering successes and innovations, many of which were learned from country-level experiences, and all of which have supported the Americas’ milestone of being declared the world’s first region to eliminate polio, rubella, congenital rubella syndrome and measles. The Region’s recent successes and innovations have included the 2003 launch of Vaccination Week in the Americas, after which other world regions followed suit; in 2012, World Immunization Week was established. This strategy of broad social participation— putting vaccines on the public agenda and aiming to leave no one behind—was identified as one of the five memorable movements in public health by the Global Development Professionals Network in the British newspaper *The Guardian*. (16) The success of the EPI depends on safe and effective vaccines, people’s confidence in vaccines, and their access to them. Success in these areas is reflected in sustained high coverage levels and the elimination and control of important communicable diseases. A range of immunization issues are analyzed in the supplement from different perspectives, including progress made and current challenges, linkages to the determinants of health, and how these determinants reflect regional inequalities. Also addressed in this edition is the process of introducing new vaccines, such as the HPV vaccine; the work to continue ensuring vaccine safety; and the use of information for better vaccine-related decision making. Finally, this supplement considers the future of immunization in the Americas and the long road ahead.

Against the backdrop of its history, successes, and challenges, the EPI stands out as an invaluable public resource, built by countless anonymous heroes including health professionals, vaccinators, community leaders, families, teachers, transport professionals, communicators, and volunteers. This supplement to the Journal recognizes their outstanding role, solidarity, leadership, sacrifice, passion, commitment, and perseverance in advancing health for all and with all. We trust that it will provide a wealth of information and inspiration for health professionals and others committed to immunization in the Americas.

## Appreciation

The Pan American Journal of Public Health recognizes with appreciation the contributions of the members of the Editorial Committee, and authors of the Overview article. Their contributions and dedication to this issue on immunization in the Region of the Americas were extraordinary and helped make the manuscripts more interesting, more accurate, and more useful to our readers and all others who work to improve the health of the peoples of the Americas.

The Journal would like to give special thanks to the General Coordination of the National Immunization Program, Department of Transmissible Disease Surveillance, Health Surveillance Secretariat, Ministry of Health, Brazil, whose financial and programmatic contributions were essential to the publication of this special issue.
